# Smartphone language features may help identify adverse post-traumatic neuropsychiatric sequelae and their trajectories

**DOI:** 10.1038/s44277-025-00028-x

**Published:** 2025-05-20

**Authors:** Lisa Vizer, Jennifer Pierce, Yinyao Ji, Meredith A. Bucher, Mochuan Liu, Lyle Ungar, Salvatore Giorgi, Zhaopeng Xing, Stacey L. House, Francesca L. Beaudoin, Jennifer S. Stevens, Thomas C. Neylan, Gari D. Clifford, Tanja Jovanovic, Sarah D. Linnstaedt, Donglin Zeng, Laura T. Germine, Kenneth A. Bollen, Scott L. Rauch, John P. Haran, Alan B. Storrow, Christopher Lewandowski, Paul I. Musey, Phyllis L. Hendry, Sophia Sheikh, Christopher W. Jones, Brittany E. Punches, Lauren A. Hudak, Jose L. Pascual, Mark J. Seamon, Erica Harris, Claire Pearson, David A. Peak, Roland C. Merchant, Robert M. Domeier, Brian J. O’Neil, Paulina Sergot, Leon D. Sanchez, Steven E. Bruce, Steven E. Harte, Ronald C. Kessler, Karestan C. Koenen, Samuel A. McLean, Xinming An

**Affiliations:** 1 Department of Medicine, Division of General Medicine and Clinical Epidemiology, University of North Carolina at Chapel Hill, Chapel Hill, NC 27559 USA; 2 Department of Anesthesiology, University of Michigan, Ann Arbor, MI 48109 USA; 3 Institute for Trauma Recovery, Department of Psychiatry, University of North Carolina at Chapel Hill, Chapel Hill, NC 27559 USA; 4 Department of Biostatistics, University of North Carolina at Chapel Hill, Chapel Hill, NC 27559 USA; 5 Computer and Information Science, University of Pennsylvania, Philadelphia, PA 19104 USA; 6 CVS Health, New York, NY 10029 USA; 7 Department of Emergency Medicine, Washington University School of Medicine, St. Louis, MO 63110 USA; 8 Department of Epidemiology, Brown University, Providence, RI 02930 USA; 9 Department of Emergency Medicine, Brown University, Providence, RI 02930 USA; 10 Department of Psychiatry and Behavioral Sciences, Emory University School of Medicine, Atlanta, GA 30329 USA; 11 Departments of Psychiatry and Neurology, University of California San Francisco, San Francisco, CA 94143 USA; 12 Department of Biomedical Informatics, Emory University School of Medicine, Atlanta, GA 30332 USA; 13 Department of Biomedical Engineering, Georgia Institute of Technology and Emory University, Atlanta, GA 30332 USA; 14 Department of Psychiatry and Behavioral Neurosciences, Wayne State University, Detroit, MI 48202 USA; 15 Institute for Trauma Recovery, Department of Anesthesiology, University of North Carolina at Chapel Hill, Chapel Hill, NC 27559 USA; 16 Institute for Technology in Psychiatry, McLean Hospital, Belmont, MA 02478 USA; 17 The Many Brains Project, Belmont, MA 02478 USA; 18 Department of Psychiatry, Harvard Medical School, Boston, MA 02115 USA; 19 Department of Psychology and Neuroscience & Department of Sociology, University of North Carolina at Chapel Hill, Chapel Hill, NC 27559 USA; 20 Department of Psychiatry, McLean Hospital, Belmont, MA 02478 USA; 21 Department of Emergency Medicine, University of Massachusetts Chan Medical School, Worcester, MA 01655 USA; 22 Department of Emergency Medicine, Vanderbilt University Medical Center, Nashville, TN 37232 USA; 23 Department of Emergency Medicine, Henry Ford Health System, Detroit, MI 48202 USA; 24 Department of Emergency Medicine, Indiana University School of Medicine, Indianapolis, IN 46202 USA; 25 Department of Emergency Medicine, University of Florida College of Medicine -Jacksonville, Jacksonville, FL 32209 USA; 26 Department of Emergency Medicine, Cooper Medical School of Rowan University, Camden, NJ 08103 USA; 27 Department of Emergency Medicine, Ohio State University College of Medicine, Columbus, OH 43210 USA; 28 Ohio State University College of Nursing, Columbus, OH 43210 USA; 29 Department of Emergency Medicine, Emory University School of Medicine, Atlanta, GA 30329 USA; 30 Department of Surgery, Department of Neurosurgery, University of Pennsylvania, Philadelphia, PA 19104 USA; 31 Perelman School of Medicine, University of Pennsylvania, Philadelphia, PA 19104 USA; 32 Department of Surgery, Division of Traumatology, Surgical Critical Care and Emergency Surgery, University of Pennsylvania, Philadelphia, PA 19104 USA; 33 Department of Emergency Medicine, Einstein Medical Center, Philadelphia, PA 19107 USA; 34 Department of Emergency Medicine, Wayne State University, Ascension St. John Hospital, Detroit, MI 48236 USA; 35 Department of Emergency Medicine, Massachusetts General Hospital, Boston, MA 02114 USA; 36 Department of Emergency Medicine, Harvard Medical School, Boston, MA 02115 USA; 37 Department of Emergency Medicine, Brigham and Women’s Hospital, Boston, MA 02115 USA; 38 Department of Emergency Medicine, Trinity Health-Ann Arbor, Ypsilanti, MI 48197 USA; 39 Department of Emergency Medicine, Wayne State University, Detroit Receiving Hospital, Detroit, MI 48202 USA; 40 Department of Emergency Medicine, McGovern Medical School at UTHealth, Houston, TX 77030 USA; 41 Department of Psychological Sciences, University of Missouri - St. Louis, St. Louis, MO 63121 USA; 42 Department of Anesthesiology, University of Michigan Medical School, Ann Arbor, MI 48109 USA; 43 Department of Internal Medicine-Rheumatology, University of Michigan Medical School, Ann Arbor, MI 48109 USA; 44 Department of Health Care Policy, Harvard Medical School, Boston, MA 02115 USA; 45 Department of Epidemiology, Harvard T.H. Chan School of Public Health, Harvard University, Boston, MA 02115 USA; 46 Department of Emergency Medicine, University of North Carolina at Chapel Hill, Chapel Hill, NC 27559 USA

**Keywords:** Diagnostic markers, Neurological manifestations

## Abstract

Language features may reflect underlying cognitive and emotional processes following a traumatic event that portend clinical outcomes. The authors sought to determine whether language features from usual smartphone use were markers associated with concurrent posttraumatic symptoms and worsening or improving posttraumatic symptoms over time following a traumatic exposure. This investigation was a secondary analysis of the Advancing Understanding of RecOvery afteR traumA study, a longitudinal study of traumatic outcomes among survivors recruited from 33 emergency departments across the United States. Adverse posttraumatic sequelae were assessed over the six months following the initial traumatic exposure. Language features were extracted from usual smartphone use in a specialized app. Bivariate linear mixed models were used to identify and validate language features that are markers associated with posttraumatic symptoms. Participants were 1744 trauma survivors, with a mean age of 39 [SD = 13] years old, and 56% were female. Fourteen language features were associated with severity level of posttraumatic symptoms at specific timepoints (cross-sectional markers) and five features were associated with change in severity level of posttraumatic symptoms (longitudinal markers). References to the body and health or illness were predictive of worsening pain, somatic, and thinking/concentration/fatigue symptom severity over time. An increase in references to others was associated with improvement in somatic symptom severity over time and increases in expressions of causation or cognitive processes were associated with improvement in pain symptom severity over time. Language features derived from usual smartphone use can convey important information about health, functioning, and recovery following a traumatic event. Clinicians might utilize such information to determine who may experience a high symptom burden or risk of worsening posttraumatic symptoms.

## Lay Summary

Via usual smartphone use following trauma exposure, this study identified language markers associated with patient-reported severity and change in severity for multiple symptoms. Using language markers as a proxy for the status of and changes in specific symptoms supports efficient remote health status monitoring and can provide clinicians with valuable real-time insights into health, functioning, and recovery. These insights can be leveraged to guide targeted interventions tailored to individual trauma survivors.

## Smartphone language features may help identify adverse post-traumatic neuropsychiatric sequelae and their trajectories

Take a Deep Dive into this article

## Introduction

Nearly 90% of US adults report exposure to at least one traumatic event during their lifetime [1], with many presenting to an emergency department (ED) for treatment after the experience. Although most are not hospitalized, they are at risk of developing adverse posttraumatic neuropsychiatric sequelae (APNS), including pain and other somatic symptoms, thinking/concentration/fatigue, depression, avoidance, re-experiencing, anxiety, hyperarousal, sleep disruption, and nightmares [2]. These posttraumatic symptoms are highly comorbid [3–5] and contribute to negative outcomes such as distress, functional impairment [6, 7], and reduced quality of life [8, 9]. However, physicians lack tools to determine which people are at risk for severe symptoms or might experience longer-lasting symptom burden, particularly as follow-up after an ED visit is often limited.

Computational linguistics has gained attention as an approach for understanding psychological aspects of human language. Combining techniques from linguistics, cognitive science, and artificial intelligence, computational linguistics facilitates automated processing and analysis of human language and is increasingly used to detect mental illness from text [10, 11]. To generate language features, word data are processed using validated models that estimate levels of psychological traits including personality [12], loneliness [13], politeness [14], depression [15], stress [16], emotion categories [17], and sentiment [18–20]. These language features may reflect underlying cognitive and emotional processes following a traumatic event that are important for clinical outcomes.

Prior work on language features after a traumatic experience centers on data gathered from either written and transcribed trauma narratives or other sources including non-trauma narratives or memories and social media posts. Previous research in the context of trauma narratives suggests that people diagnosed with posttraumatic stress disorder (PTSD) use higher rates of first-person singular pronouns [21], sensory words (e.g., sight, sound, taste) [22], and emotion words (e.g., anxiety, sadness) [23, 24]. Studies examining symptom severity related to trauma narratives find that lower rates of words expressing cognitive processes (e.g., thoughts, insights) [22, 25], words concerned with death and dying [26], and shorter narrative length [27, 28] predict poorer outcomes. However, a risk of participant distress is inherent in using trauma narratives as data [29].

Text generated through non-trauma narratives and memories or shared on social media offers a more naturalistic dataset than restricting text to trauma narratives. Prior research has shown that the results from studies with trauma narrative text are largely replicated in studies with social media text [30]. Fewer positive and more negative emotion words were associated with more severe avoidance and numbing symptoms [31, 32]. A PTSD diagnosis was associated with higher use of first-person singular pronouns and lower use of first-person plural pronouns [33, 34]. Findings on cognitive processing words are inconsistent [30]. Studies using non-trauma narratives and memories are less naturalistic but usually include well-characterized participants. Although more naturalistic, difficulties verifying diagnoses, symptoms, and demographics for social media users are a consideration in evaluating the outcomes of these studies. This study avoids the shortcomings of other data sources by using the words from usual smartphone use. Our approach avoids the potential distress associated with trauma narratives, provides more naturalistic text than non-trauma narratives, and ensures better characterized participants than social media text.

The vast majority of studies only include the umbrella PTSD diagnosis [21–28, 33, 34] or only a small number of more specific APNS symptom domains [31, 32]. As demonstrated in other examinations of language and co-occurring conditions [35], investigating PTSD without considering individual APNS symptoms domains misses the opportunity to disambiguate language features that are common to more than one APNS or specific to only one. This disambiguation allows better classification and informs transdiagnostic treatments as well as symptom-specific interventions. To understand links between smartphone language features and specific APNS symptom domains, this study examines associations between language features and scores for ten symptom domains.

Leveraging the near ubiquity of smartphones to advance current knowledge of the relationship between usual language and posttraumatic symptoms, we generated language features from smartphone word data and analyzed them against self-reported APNS symptoms from a diverse population of adults (*n* = 1744) presenting to an ED after traumatic stress exposure. We identified and internally validated language features as both cross-sectional, between-subjects markers (i.e., language characteristics that indicate high concurrent symptoms) and longitudinal, within-subjects markers (e.g., language characteristics that predict worsening or improving symptoms over time). Markers with cross-sectional between-subjects associations can help differentiate severity levels of APNS symptoms at a point in time. Those with longitudinal within-subjects associations can help differentiate worsening or improving APNS symptoms over time.

## Materials and methods

### Study overview and sample characteristics

This investigation was a secondary analysis of the AURORA (Advancing Understanding of RecOvery afteR traumA) study [2]. AURORA collected prospective genomic, neuroimaging, psychophysical, physiological, neurocognitive, digital phenotype, and self-report data from a diverse sample of trauma survivors who visited one of 33 EDs within 72 h of their trauma. The full methodology of the AURORA study is described in detail by McLean et al. [2] and was ethically approved by the Institutional Review Board (IRB #17-0703) at UNC Chapel Hill. Beginning in September 2017 AURORA recruited participants who were aged 18–65 years old, able to speak and read English, able to follow the protocol at the time of enrollment, physically able to use a smartphone, expected that they would have access to a smartphone for at least one year following study enrollment, and had possessed a smartphone for at least one year prior to study enrollment. We excluded patients if they had a solid organ injury Grade > I according to the American Association for the Surgery of Trauma Injury Scoring Scales [36], significant hemorrhage, needed a chest tube or surgery with anesthesia, or were likely be admitted for > 72 h. Qualifying traumatic events included motor vehicle collision, physical assault, sexual assault, falls > 10 feet, or mass casualty incidents. Although the aim of the AURORA study was to record data from 5000 individuals over five years, data are being analyzed periodically to report early results to the scientific community. This work analyzes data through July 2020 from 1744 participants who used Android smartphones. We did not include participants with Apple phones due to Apple’s privacy restrictions. All participants provided written informed consent after receiving a complete description of the study. By excluding trauma survivors with long bone fractures, laceration with significant hemorrhage, and solid organ injury, the AURORA study was designed to disentangle the potential influence of physiological effects related to general anesthesia, hemorrhage and medication on these APNS symptom development.

### Survey data collection and APNS symptom score generation

The Mindstrong Discovery™ app was installed onto the participants’ Android smartphone during enrollment to prompt participants to complete brief “flash” surveys assessing APNS domains at 10 or 11 timepoints. This investigation used flash survey and word data collected in the first six months following enrollment and focuses on ten self-reported measures of psychological and physical symptoms associated with APNS: pain [37, 38], depression [39–42], sleep continuity [43], nightmares [44–46], somatic symptoms [37, 47], thinking/concentration/fatigue [48–51], avoidance, re-experiencing, anxiety [52, 53], and hyperarousal [54–57]. For each of the ten APNS symptom domains, flash survey items were selected by domain experts as indicator variables to develop joint measurement models across all timepoints based on confirmatory factor analysis. Flash survey items used to define the 10 symptoms and the days on which each was administered are provided in Appendix 1. The joint measurement model pooled data across all timepoints to estimate a consistent set of latent factors (symptom domains) where all flash survey items relevant to the APNS symptom domain were included as indicator variables. Factor scores for each symptom were computed for each participant for each timepoint as measures to define these 10 APNS symptom domains. These factor scores were then used to identify and validate language markers that are associated with specific APNS symptom domains either at a point in time (cross-sectional between-subjects trait markers) or over time (longitudinal within-subjects state markers).

### Word data collection and language feature generation

The Mindstrong Discovery™ app continuously and passively collected all words entered on the smartphone via the native keyboard or a Mindstrong keyboard and recorded an unordered list of unique words and their frequencies each day. Reconstructing individual text messages, emails, or other text-based interactions from this list is impossible, ensuring participant privacy. These word data were de-identified and encrypted to further ensure confidentiality. As in other studies of language in the context of mental health [35], we then generated language-based estimates of psychological traits and lexicon-based characteristics of text. Validated language models of emotion calculated estimated levels of psychological traits such as personality [12], loneliness [13], politeness [14], depression [15], stress [16], emotion categories [17], and sentiment [18–20]. Language Inquiry and Word Count (LIWC) software [58] calculated lexical characteristics such as frequency of parts of speech, references to other people, and word count (see all characteristics in Appendix 3). All language features were normalized to introduce a common scale and ensure that higher scores indicate a higher level of the modeled attribute, and lower scores indicate a lower level of the attribute.

### Language feature preprocessing, identification, internal validation, and evaluation

We preprocessed the normalized language features generated in the prior step by checking for extreme and abnormal values, missing and zero percentage of each language feature, as well as the correlation between each pair of features. Next, we identified subsets of language features that were highly correlated (Spearman correlations > 0.85) to reduce redundancy. For each subset, we retained the feature that showed the strongest univariate association with APNS symptom scores or was deemed conceptually most interpretable based on prior research. To minimize the inclusion of features with limited variability, we excluded language features with a zero percentage larger than 90%, set word counts larger than 10,000 to missing, and removed participants who switched smartphone operating system (ex. Android to Apple iOS) within the first six months. Word counts greater than 10,000 were set to missing to address potential outliers that could skew the results. Such extreme values likely reflect data collection artifacts or atypical user behavior, and their exclusion is intended to enhance the robustness of the analysis.

Because missing data is common for large-scale, longitudinal, naturalistic studies, it is critical to examine the missing mechanism of these missing values. We calculated the correlations of the APNS symptom scores with the completion rate of the four main study activities–survey, flash survey, neurocognitive tests, and watch wearing–to evaluate whether missing values were associated with symptom severity. Identifying these associations helps determine whether missingness in the data may be systematically related to participants’ clinical characteristics, which is critical for interpreting results and addressing potential biases in the analysis. All the correlations are weak (<0.1, Appendix 2), suggesting that missing data did not bias the main outcome associations. As a result, we considered these missing data as missing at random, and these missing values were handled by the bivariate linear mixed model using full information maximum likelihood estimation method.

For each flash survey timepoint for each participant, the mean of the language features from the day prior to and the day of APNS symptom data collection were merged with each of the 10 APNS symptom scores and used as candidate language markers for corresponding APNS symptom domains. We randomly divided the aggregated data into two equal parts and used one half for marker identification and the other half for internal validation. The internal validation step was applied to ensure that the markers were consistently associated with the symptoms. To identify and validate markers, we estimate and test the correlation between language features and each of the 10 symptom domains with repeated measures from each participant. To account for the correlation structure of the repeated measures, we used a bivariate linear mixed model approach [59, 60] to simultaneously model the cross-sectional and longitudinal associations of each language feature within each symptom domain. Using the first half of the data, language feature variables are identified as potential language markers if their associations (either cross-sectional or longitudinal) with any of the 10 APNS symptom domains are statistically significant (adjusted *p*-value < 0.05) after False Discovery Rate (FDR) multiple tests correction. These potential markers are then further validated using the same bivariate linear mixed model on the remaining 50% of the data, where markers retaining statistically significant associations (adjusted *p*-value < 0.05) after Bonferroni multiple tests correction are confirmed as markers for corresponding APNS symptom domains. For those longitudinal language markers passing both identification and validation steps, we evaluated for their accuracy in predicting change in the corresponding APNS symptom scores (e.g., worsening versus improvement). A simple cut-off was used to define worsening and improvement, such that worsening of symptom severity is defined as *(severity score at six months minus severity score at one week) > 0*, and improvement in symptom severity is defined as *(severity score at six months minus severity score at one week) < 0*. The predicted symptom score change was generated by applying the same cut-offs to the change of longitudinal markers over the same time window. The sensitivity, specificity, and positive and negative predictive value (PPV, NPV) of the change in marker value associated with the change in symptom score were then assessed. High PPV for worsening or high NPV for improvement would suggest that language markers derived from smartphone use may have utility as initial screening measures for adverse posttraumatic outcomes among trauma survivors.

## Results

### Sample sociodemographic, trauma exposure, and clinical characteristics

**Fig. 1:**
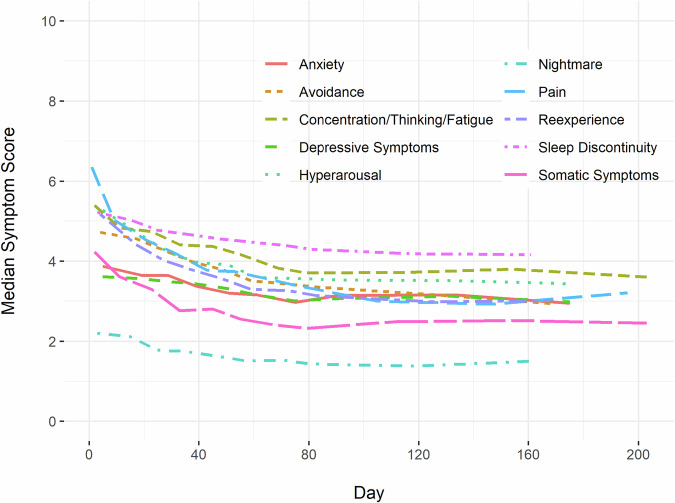
Median adverse posttraumatic neuropsychiatric symptom scores over the first 6 months after trauma exposure.

Demographic characteristics of the participants are presented in Table 1. Most of the participants were female (55.5%) with a mean age of 39.0 years old (SD 13.0). More than half were non-Hispanic Black (53.6%), followed by non-Hispanic White (31.7%), and Hispanic (10.9%). The majority did not have a college degree (84.4%). The traumatic exposure that conferred eligibility for the study was a motor vehicle collision for almost three-quarters of the sample (71.0%). Median symptom scores over the first 6 months after trauma exposure indicate that moderate symptoms (e.g., pain > = 4 on a scale of 0–10) were observed for much of the sample and symptom burden tended to improve over time (Fig. 1).

**Table 1:** Demographic information.

*N* = 1744		
**Age (y) (Mean, SD)**	39.0 (13.0)	
Gender	n	%
Female	968	55.5
Race	n	%
Hispanic	190	10.9
Non-Hispanic White	553	31.7
Non-Hispanic Black	935	53.6
Non-Hispanic Other	58	3.3
Education Status	n	%
High school or less	262	15.0
High school graduate	496	28.4
Some college	715	41.0
College or more	264	15.1
Marital Status	n	%
Married	364	20.9
Separated/Divorced/Widowed/Annulled	388	22.2
Never been married	978	56.1
Trauma Type	n	%
Motor Vehicle Collision	1238	71.0
Physical Assault	204	11.7
Sexual Assault	7	0.4
Fall > = 10 feet	29	1.7
Incident causing traumatic stress exposure to many people	7	0.4
Non-motorized Collision	25	1.4
Fall < 10 feet or from unknown height	104	6.0
Burns	11	0.6
Animal-related	45	2.6
Other	74	4.2

### Language feature characteristics

All participants used Android smartphones for the duration of the study and typed a mean of 387.13 (SD = 1137.43) and a median of 143 words per day. Summary statistics for the language features are in Appendix 3, including examples of words associated with each language feature.

### Cross-sectional language markers

We first identified and internally validated cross-sectional language markers that differentiated individuals experiencing different severities of specific APNS symptoms at any point in time. Fourteen language markers met internal validation criteria for seven APNS symptom domains with some markers associated with more than one symptom domain. The significant cross-sectional, between-subjects correlations between symptom severity scores and language markers are reported in Table 2. We internally validated four significant cross-sectional language markers for the severity of pain, nine for somatic symptoms, one for avoidance, three for hyperarousal, two for nightmares, two for depression, and one for anxiety. Higher levels of pain and somatic symptoms were associated with more frequent references to people (family, other people, males). Higher levels of pain were associated with less frequent expressions of tentativeness and more frequent expressions of happiness. Higher levels of somatic symptoms and hyperarousal were associated with more frequent expressions of loneliness and less frequent use of articles. Higher levels of somatic symptoms, nightmares, and avoidance were associated with more frequent use of first-person singular pronouns and higher levels of somatic symptoms and nightmares were associated with less frequent use of longer words (>6 letters). Higher levels of hyperarousal, depression, and anxiety were associated with more frequent expressions of negative emotion. Lastly, higher levels of somatic symptoms were associated with more frequent use of all personal pronouns and informal speech.

**Table 2:** Smartphone language markers (average of two 24-h periods) associated with cross-sectional adverse posttraumatic neuropsychiatric (APNS) symptom severity

APNS symptom	Language marker	Correlation	*P*-value	Adjusted *p*-value^a^
Pain	References to family^b^	0.2560	<0.001	0.0088
	References to other people^b^	0.2467	<0.001	0.0088
	Expressions of tentativeness^b^	−0.2371	<0.001	0.0088
	Expressions of happiness^c^	0.1805	<0.001	0.0352
Somatic Symptoms	Use of first-person singular pronouns^b^	0.2660	<0.001	0.0088
	Use of any personal pronouns^b^	0.1747	<0.001	0.0352
	References to males^b^	0.2711	<0.001	0.0088
	References to other people^b^	0.2445	<0.001	0.0088
	Words containing over six letters^b^	−0.2326	<0.001	0.0088
	Expressions of tentativeness^b^	−0.2343	<0.001	0.0088
	Use of informal speech^b^	0.1761	<0.001	0.0264
	Use of articles^b^	−0.1889	<0.001	0.0352
	Expressions of loneliness^d^	0.2562	<0.001	0.0088
Avoidance	Use of first-person singular pronouns^b^	0.1926	<0.001	0.0088
Hyper-arousal	Expressions of negative emotions^b^	0.2840	<0.001	0.0088
	Use of articles^b^	−0.2422	<0.001	0.0176
	Expressions of loneliness^d^	0.2319	<0.001	0.0088
Nightmare	Use of first-person singular pronouns^b^	0.2410	<0.001	0.0088
	Words containing over six letters^b^	−0.1886	<0.001	0.0176
Depression	Expressions of negative emotion^b^	0.3360	<0.001	0.0088
	Expressions of anger^b^	0.1723	<0.001	0.0264
Anxiety	Expressions of sadness^b^	0.6513	0.0002	0.0176

^a^Bonferroni adjusted *p*-values are used.

^b^Derived using Language Inquiry and Word Count (LIWC) software.

^c^Derived using Dodds, [18].

^d^Derived using Guntuku, [13, 16].

All values are from the validation sample.

### Longitudinal language markers

We next identified and internally validated longitudinal language markers whose changes are associated with changes in severity score (either worsening or improvement) of specific APNS symptom domains. Five language markers met internal validation criteria for three APNS symptom domains with some markers associated with more than one symptom domain. The statistically significant longitudinal, within-subjects correlations between change in APNS symptom scores and change in language markers are reported in Table 3. We internally validated four significant language longitudinal markers for change in severity of pain, two for somatic symptoms, and one for thinking/concentration/fatigue. Increases in pain, somatic symptoms, and problems with thinking/concentration/fatigue were associated with an increase in references to health or illness, while an increase in pain was also associated with an increase in references to the body. A decrease in pain was associated with an increase in references to causation and cognitive processes. Finally, a decrease in somatic symptoms was associated with an increase in references to other people.

**Table 3:** Changes in smartphone language markers associated with longitudinal changes in adverse posttraumatic neuropsychiatric (APNS) symptom severity

APNS symptom	Language marker^a^	Correlation	*P*-value	Adjusted *p*-value^b^
Pain	References to the body	0.0684	<0.001	0.0023
	References to health or illness	0.1303	<0.001	0.0023
	Expressions of causation	−0.0527	0.0013	0.0299
	Expressions of cognitive processes	−0.0523	0.0015	0.0345
Somatic Symptoms	References to health or illness	0.0585	<0.001	0.0023
	References to other people	−0.0621	<0.001	0.0023
Thinking/Concentration/Fatigue	References to health or illness	0.0573	<0.001	0.0046

^a^Derived using Language Inquiry and Word Count (LIWC) software.

^b^Bonferroni adjusted *p*-values are used.

All values are from the validation sample.

#### Utility of predicting worsening and improvement using longitudinal language markers

We evaluated the potential utility of the longitudinal language markers for predicting worsening and improvement of symptoms from one week to six months post-trauma. Worsening pain severity was experienced by 24% of participants, worsening somatic symptom severity was experienced by 22% of participants, and worsening thinking/concentration/fatigue severity was experienced by 28% of participants. PPV was between 0.76 and 0.82 for all markers (see Table 4). Improvement in pain severity was experienced by 76% of participants, improvement in somatic symptom severity was experienced by 78% of participants, and improvement in thinking/concentration/fatigue severity was experienced by 72% of participants. In general, the prediction of improvement in severity was slightly less accurate for each marker than the prediction of worsening severity. NPV was between 0.68 and 0.78 for all markers (see Supplementary Table 1). Relatively high PPV for symptom worsening and High NPV for symptom improvement indicate that these longitudinal markers can predict symptom worsening better than improvement, based on this simple prediction model. However, no single marker achieved both high PPV and NPV in predicting the change of APNS symptom scores.

**Table 4:** Prediction of worsening adverse posttraumatic neuropsychiatric (APNS) symptom severity using smartphone language markers.

APNS symptom	N of worsening participants	% of worsening participants	Language marker^a^	Sensitivity	Specificity	PPV	NPV
Pain	186	24%	References to the body	0.49	0.51	0.77	0.24
	186	24%	References to health or illness	0.58	0.42	0.77	0.23
	186	24%	Expressions of causation	0.68	0.34	0.78	0.24
	186	24%	Expressions of cognitive processes	0.62	0.38	0.76	0.24
Somatic Symptoms	170	22%	References to health or illness	0.60	0.50	0.82	0.26
	170	22%	References to other people	0.49	0.51	0.78	0.22
Thinking/Concentration/Fatigue	217	28%	References to health or illness	0.61	0.48	0.76	0.32

*PPV* positive predictive values, *NPV* negative predictive values.

^a^Derived using Language Inquiry and Word Count (LIWC) software.

## Discussion

Using language data collected from usual smartphone use, we derived and internally validated 14 cross-sectional and five longitudinal relationships between language features and APNS symptom severity during the first six months following a traumatic event – a high-risk period during which trauma survivors might experience either symptom recovery or persistence [2]. To our knowledge, this is the first study to examine such associations using word data from usual smartphone use. Of interest, pain and somatic symptoms exhibited several cross-sectional and longitudinal associations, suggesting that APNS symptom domains not traditionally considered part of a PTSD diagnosis may be uniquely associated with language features.

In particular, higher frequency of references to others (i.e., family, other people, males) was associated in the cross-sectional analysis with higher pain and somatic symptom scores and an increase in frequency in references to others was associated in the longitudinal analysis with a decrease in somatic symptom scores. The paradox of both higher frequency of references to others being associated with *higher* somatic symptom severity and increased frequency of references to others being associated with *decreased* symptom severity should be further investigated. In addition, higher frequency of expressions of loneliness was associated with higher hyperarousal scores. These results concerning references to others and expressions of loneliness support a link between social processes and health outcomes, as suggested by prior work [61], and the role of language should be investigated further. Also, in accordance with previous research, higher scores in the somatic, avoidance, and nightmare domains were associated with higher frequency of use of first-person singular pronouns [21, 22, 62]. This finding may suggest an understandable focus on the self in response to posttraumatic symptoms and may constitute a useful health status marker.

Similar to previous research findings [22, 26, 63–65], more frequent use of indicators of language complexity such as words containing over six letters, articles, and expressions of tentativeness were associated with lower cross-sectional scores for pain, somatic, hyperarousal, and nightmare symptom domains. More frequents expressions of sadness, anger, and negative emotions were associated only with higher scores in the affective domains of hyperarousal and depression, and were consistent with prior research suggesting that those with PTSD tend to express more sadness and less happiness [66]. However, in our sample, more frequent expressions of happiness were associated with higher scores in the pain symptoms domain, supporting the notion that APNS symptom domains may have unique or overlapping language marker profiles and necessitating further investigation.

We found that worsening pain, somatic, and thinking/concentration/fatigue APNS symptom domain scores were associated with more frequent references to the body and health or illness. As with the use of first-person singular pronouns, this usage increase may indicate an understandable increase in focus on concerning APNS symptoms, but prior research has not evaluated language use associations with these specific domains so further work is needed.

The stronger correlations observed in Table 2 (cross-sectional associations) compared to Table 3 (longitudinal associations) likely reflect the immediate relationship between smartphone usage metrics and contemporaneous survey responses. This suggests that smartphone behavior may be more tightly linked to psychological states or experiences at the moment rather than longitudinally over time.

Smartphones are a near ubiquitous vehicle for communication and internet access. The words we use during interactions mediated by smartphones can convey information about our health, including symptoms occurring after a traumatic experience. In the future, language markers may help clinicians identify individuals who are experiencing a high level of symptom burden or who are at risk for worsening symptoms and merit further evaluation for adverse outcomes. These same language markers might also be useful for monitoring response to treatments or therapeutic interventions.

## Limitations

Several limitations are relevant when considering the results of this investigation. Our participants were enrolled in the ED, thus generalizability of findings to trauma survivors who do not present to the ED is not known. The majority of participants were survivors of motor vehicle collisions and the generalizability of results to other types of trauma is also not known. Although missing data were not correlated with the study outcome measures, other missingness patterns could not be accounted for in the analysis. We also used data only from Android phone users and their use patterns may vary from users of other smartphones. We acknowledge that administering flash surveys may have inadvertently primed participants, influencing their word usage or psychological responses. For example, completing a survey in the morning could subtly shape participants’ smartphone interactions later in the day. Furthermore, the daily administration of flash surveys, sometimes on consecutive days, could introduce overlap or carryover effects in word usage or psychological states. For instance, language metrics tied to one construct might inadvertently capture signals from related constructs measured on adjacent days. Future studies are needed to disentangle potential priming effect from natural variation in language usage and psychological symptoms and to adjust for temporal dependencies.

We also remind readers that this investigation is exploratory rather than being a confirmatory study testing theoretical hypotheses. Additionally, study assessments were limited to the six months following a traumatic event and only used language data. Future studies might investigate generalizability and should examine associations between APNS symptom domains and language features in conjunction with other passive data, such as keystroke and activity data, collected over longer durations and including sex-related differences. While our primary analyses did not specifically examine sex-related differences in smartphone word use, this is an important and relevant area for further analyses to assess whether sex might moderate the relationship between language usage and APNS symptoms after trauma exposure. Future studies could leverage symptom trajectory data to investigate whether patterns of smartphone language use vary based on longitudinal symptom trajectories. Finally, we want to note that when demonstrating the potential utility of predicting symptom changes (both worsening and improvement) using longitudinal language markers, we employed a very simple prediction model based solely on individual markers. Consequently, the overall prediction accuracy was not high. We anticipate that much better prediction accuracy can be achieved by using more advanced statistical and machine learning techniques, which would combine all the longitudinal markers along with baseline characteristics. We also recognize that the small degree of change defined by the cutoff of zero may not represent clinically meaningful shifts. This decision was made partly due to a lack of widely accepted cutoffs for these symptoms. However, these improvements are beyond the scope of the current study.

## Conclusion

This investigation sought to establish the relationship between language features derived from usual smartphone use and the severity and change in posttraumatic symptoms. The use of smartphone interactions as the data source after a traumatic experience rather than trauma narratives is unique and avoids additional patient burden and possible distress. We identified fourteen language markers for cross-sectional severity of seven APNS symptom domains and five language markers for longitudinal changes in severity for three APNS symptom domains. These findings confirm that language markers derived from usual smartphone use convey important information about health, functioning, and recovery following a traumatic event. Patterns of language use could contribute to remote symptom monitoring during the post trauma period and clinicians might use such information to identify those at risk for high symptom burden or worsening posttraumatic symptoms. Future research includes investigating sex differences and novel associations.

Data used in this manuscript is available through the National Institute of Mental Health (NIMH) Data Archive (NDA). The NDA Collection for the AURORA Project can be found here: https://nda.nih.gov/edit_collection.html?id=2526.

## Supplementary information

Supplemental Information
