# Supplementary material for: Smartphone language features may help identify adverse post-traumatic neuropsychiatric sequelae and their trajectories
Source: NPP Digit Psychiatry Neurosci. 2025 May 20;3:8. doi: 10.1038/s44277-025-00028-x (PMC12092297; doi:10.1038/s44277-025-00028-x)
Supplement: Supplementary file 1 — Supplemental Information [file 44277_2025_28_MOESM1_ESM.docx]

**Supplemental Information**

**Supplemental Table 1. Prediction of improvement in adverse posttraumatic neuropsychiatric (APNS) symptom severity over time using smartphone language biomarkers**

| **APNS symptom** | **N of improved participants** | **% of improved participants** | **Language biomarker*** | **Sensitivity** | **Specificity** | **PPV** | **NPV** |
| --- | --- | --- | --- | --- | --- | --- | --- |
| Pain | 599 | 76% | References to the body | 0.49 | 0.51 | 0.23 | 0.76 |
|  | 599 | 76% | References to health or illness | 0.58 | 0.42 | 0.23 | 0.77 |
|  | 599 | 76% | Expressions of causation | 0.66 | 0.32 | 0.22 | 0.76 |
|  | 599 | 76% | Expressions of cognitive processes | 0.62 | 0.38 | 0.24 | 0.76 |
| Somatic Symptoms | 611 | 78% | References to health or illness | 0.50 | 0.40 | 0.19 | 0.74 |
|  | 611 | 78% | References to other people | 0.49 | 0.51 | 0.22 | 0.78 |
| Thinking/Concentration/Fatigue | 565 | 72% | References to health or illness | 0.52 | 0.39 | 0.25 | 0.68 |

*Derived using Language Inquiry and Word Count (LIWC) software

Note: PPV: Positive Predictive Values; NPV: Negative Predictive Values

**Appendix 1: Questions measuring adverse posttraumatic neuropsychiatric (APNS) symptoms in smartphone flash surveys and days administered**

| **APNS symptom** | **Timepoints (Day administered during first 6 months)** | **Questions** | **Response options** |
| --- | --- | --- | --- |
| **Pain** | 1,9,21,31,43,53,  67,77,105,147,196 | 1. How would you rate your pain in the past 24 hours at its worst? 2. How would you rate your pain in the past 24 hours on average? | No pain Severe pain  0 1 2 3 4 5 6 7 8 9 10 |
| **Depression** | 5,19,29,39,51,61,  75,91,133,175 | 1. Over the past 24 hours, how often did you feel down on yourself, no good, or worthless? 2. Over the past 24 hours, how often did you feel sad depressed, or empty? 3. Over the past 24 hours, how often did you have trouble experiencing positive feelings? (for example, being unable to feel happiness or having loving feelings for people close to you) | Never Rarely Sometimes Often Very often  [0] [1] [2] [3] [4] |
| **Sleep Disturbance** | 3,15,25,35,47,57,  71,81,119,161 | 1. Over the last few nights, how much of a problem have you had falling asleep? 2. Over the last few nights, how much of a problem have you had staying asleep all night? 3. Over the last few nights, how much of a problem have you had waking up too early in the morning? | None A little Some A lot Extremely  [0] [1] [2] [3] [4] |
| **Nightmares** | 3,15,25,35,47,57,  71,81,119,161 | 1. Over the last few nights, how much of a problem have you had with nightmares or bad dreams about the event? 2. Over the last few nights, how much of a problem have you had with nightmares or bad dreams about other things? 3. Over the last few nights, how much of a problem have you had with panic attacks during the night? | None A little Some A lot Extremely  [0] [1] [2] [3] [4] |
| **Anxiety** | 5,19,29,39,51,61,  75,91,133,175 | 1. Over the past 24 hours, how often did you have severe anxiety or panic? 2. Over the past 24 hours, how often did you feel very nervous, worried, or anxious? | Never Rarely Sometimes Often Very often  [0] [1] [2] [3] [4] |
| **Hyperarousal** | 5,19,29,39,51,61,  75,91,133,175 | 1. Over the past 24 hours, how often were you “superalert” or watchful, or on guard? 2. Over the past 24 hours, how often did you feel jumpy or easily startled? | Never Rarely Sometimes Often Very often  [0] [1] [2] [3] [4] |
| **Avoidance** | 4,17,27,37,49,59,  73,83,126,168 | 1. Over the past 24 hours, how often did you avoid memories, thoughts, or feelings related to the event? 2. Over the past 24 hours, how often did you avoid external reminders of the event? (e.g., people, places, conversations, or activities) | Never Rarely Sometimes Often Very often  [0] [1] [2] [3] [4] |
| **Re-experiencing** | 4,17,27,37,49,59,  73,83,126,168 | 1. Over the past 24 hours, how often did you have repeated, disturbing, and unwanted memories of the event? 2. Over the past 24 hours, how often did you feel very upset when something reminded you of the event? 3. Over the past 24 hours, how often did you have strong physical reactions when something reminded you of the event, like heart pounding, trouble breathing, or sweating? | Never Rarely Sometimes Often Very often  [0] [1] [2] [3] [4] |
| **Somatic Symptoms** | 2,11,23,33,45,55,  69,79,112,154,203 | 1. Over the past 24 hours, how much of a problem have you had with headaches? 2. Over the past 24 hours, how much of a problem have you had with dizziness? 3. Over the past 24 hours, how much of a problem have you had with nausea? | No problem Severe problem  0 1 2 3 4 5 6 7 8 9 10 |
| **Thinking/**  **Concentration/Fatigue** | 2,11,23,33,45,55,  69,79,112,154,203 | 1. Over the past 24 hours, how much of a problem have you had with fatigue? 2. Over the past 24 hours, how much of a problem have you had concentrating? 3. Over the past 24 hours, how much of a problem have you had taking longer to think? | No problem Severe problem  0 1 2 3 4 5 6 7 8 9 10 |

**Appendix 2: Correlation of adverse posttraumatic neuropsychiatric (APNS) symptoms with completion rates of four main study tasks.**

| **APNS symptom** | **Survey** | **Verily Watch** | **Neurocognitive Tests** | **Flash Survey** |
| --- | --- | --- | --- | --- |
| Pre-Trauma Pain | .0169 | 0.037 | 0.011 | 0.011 |
| Pre-Trauma Depression | -.0167 | -0.007 | -0.021 | -0.011 |
| Pre-Trauma PTSD | -.0194 | -0.031 | -0.069** | -0.051* |
| Pre-Trauma Somatic | -.0284 | -0.014 | -0.020 | -0.022 |
| Peritraumatic Pain | -.0131 | -0.052 | -0.020 | -0.027 |
| Peritraumatic Somatic | -.0263 | -0.033 | -0.011 | -0.029 |
| Week 2 Pain | .051^*^ | .062^*^ | 0.032 | 0.046* |
| Week 2 Depression | .0095 | -0.009 | 0.012 | 0.017 |
| Week 2 PTSD | .0266 | 0.003 | 0.016 | 0.014 |
| Week 2 Somatic | -.0019 | 0.003 | -0.010 | -0.030 |
| Week 8 Pain | .0211 | 0.070** | 0.014 | 0.054* |
| Week 8 Depression | -.0010 | -0.006 | 0.016 | 0.016 |
| Week 8 PTSD | .0059 | 0.003 | 0.021 | 0.024 |
| Week 8 Somatic | -.0032 | 0.035 | 0.005 | 0.006 |
| * Correlation is significant at the 0.05 level (2-tailed).  ** Correlation is significant at the 0.01 level (2-tailed). | | | |  |
|  | | | |  |

**Appendix 3: Summary statistics for daily smartphone language features among Android users**

| **Language feature** | **Examples (N/A where not available)** | **Mean (SD)** | **Median** | | **Min** | **Max** | **Zero Percentage** |  |
| --- | --- | --- | --- | --- | --- | --- | --- | --- |
| Agreeableness^44^ | picture, cool, nice | 0.07 (1.67) | 0.03 | | -31.60 | 44.52 | 0.00 |  |
| Anger^46^ | N/A | 0.06 (0.04) | 0.05 | | 0.00 | 1.05 | 0.00 |  |
| Anticipation^46^ | N/A | 0.06 (0.05) | 0.04 | | 0.00 | 1.30 | 0.00 |  |
| Anxiety^42^ | worried, afraid | 2.58 (1.71) | 2.67 | | -33.01 | 54.85 | 0.00 |  |
| Cohens stress^43^ | depressed, pain, trying | 0.01 (1.59) | 0.05 | | -29.03 | 57.08 | 0.00 |  |
| Conscientiousness^39,44^ | calm, mean, pity | 0.14 (1.74) | 0.16 | | -31.79 | 33.35 | 0.00 |  |
| Depression^42^ | depressed why, hate | 2.08 (1.73) | 2.19 | | -23.57 | 39.91 | 0.00 |  |
| Disgust^46^ | N/A | 0.06 (0.04) | 0.05 | | 0.00 | 1.28 | 0.00 |  |
| Extraversion^39^ | amazing, party, love | 0.14 (1.82) | 0.16 | | -47.91 | 34.04 | 0.00 |  |
| Fear^46^ | N/A | 0.05 (0.04) | 0.04 | | 0.00 | 1.15 | 0.00 |  |
| Joy^46^ | N/A | 0.07 (0.07) | 0.05 | | 0.00 | 1.79 | 0.00 |  |
| Happiness^45^ | love, party, Saturday | 2.28 (0.8) | 2.12 | | 0.10 | 8.42 | 0.00 |  |
| Loneliness^40^ | myself, never, want | 0 (0) | 0.00 | | -0.01 | 0.01 | 0.00 |  |
| Negative sentiment^46^ | angry, sad | -0.22 (0.1) | -0.21 | | -5.00 | 0.00 | 0.00 |  |
| Neuroticism^39,44^ | happy, anxious, irate | 0.55 (1.6) | 0.50 | | -37.07 | 27.28 | 0.00 |  |
| Openness^39,44^ | peace, appreciate | 0.08 (1.81) | 0.06 | | -45.09 | 41.73 | 0.00 |  |
| Politeness^41^ | please, thanks, friends | 0.37 (0.22) | 0.35 | | -3.35 | 3.09 | 0.00 |  |
| Positive sentiment^46^ | joy, trust | 0.17 (0.09) | 0.15 | | 0.00 | 3.04 | 0.00 |  |
| Sadness^46^ | N/A | 0.03 (0.04) | 0.03 | | 0.00 | 1.62 | 0.00 |  |
| Surprise^46^ | N/A | 0.05 (0.04) | 0.05 | | 0.00 | 1.13 | 0.00 |  |
| Trust^46^ | N/A | 0.05 (0.04) | 0.04 | | 0.00 | 1.92 | 0.00 |  |
| **Language Metrics** | | | | | | | | |
| Word count |  | 357.21 (560.32) | 173.33 | 1.00 | | 9543.00 | 0.00 | |
| Words containing >6 letters | happiness, inability | 9.93 (6.06) | 9.29 | 0.00 | | 100.00 | 3.80 | |
| Dictionary words | like, able, oven | 292.33 (57.89) | 306.88 | 0.00 | | 700.00 | 0.57 | |
| **Function words** | it, to, no, very | 43.53 (12.24) | 46.47 | 0.00 | | 100.00 | 1.80 | |
| Total pronouns | I, them, itself | 13.57 (5.97) | 13.82 | 0.00 | | 100.00 | 3.44 | |
| Personal pronouns | I, them, her | 9.15 (4.98) | 8.94 | 0.00 | | 100.00 | 4.48 | |
| first person singular | I, me, mine | 3.01 (2.91) | 2.57 | 0.00 | | 100.00 | 9.88 | |
| first person plural | we, us, our | 0.63 (1.23) | 0.37 | 0.00 | | 100.00 | 31.01 | |
| second person | you, your, thou | 3.76 (3.54) | 3.18 | 0.00 | | 100.00 | 9.51 | |
| third person singular | she, her, him | 1.27 (1.62) | 0.87 | 0.00 | | 54.55 | 23.95 | |
| third person plural | they, their, they’d | 0.48 (0.81) | 0.27 | 0.00 | | 50.00 | 35.36 | |
| Impersonal pronouns | it, it’s, those | 4.41 (2.94) | 4.40 | 0.00 | | 66.67 | 8.20 | |
| Articles | a, an, the | 2.25 (2.03) | 2.02 | 0.00 | | 100.00 | 12.30 | |
| Prepositions | to, with, above | 10.12 (4.49) | 10.43 | 0.00 | | 100.00 | 4.09 | |
| Auxiliary verbs | am, will, have | 7.89 (4.19) | 7.92 | 0.00 | | 100.00 | 5.32 | |
| Common adverbs | very, really | 5.69 (3.35) | 5.78 | 0.00 | | 100.00 | 6.67 | |
| Conjunctions | and, but, whereas | 5.1 (3.37) | 5.00 | 0.00 | | 100.00 | 7.73 | |
| Negations | no, not, never | 1.67 (2.25) | 1.34 | 0.00 | | 100.00 | 16.21 | |
| **Grammar** | | | | | | | | |
| Regular verbs | eat, come, carry | 17.38 (6.54) | 18.25 | 0.00 | | 100.00 | 3.16 | |
| Adjectives | free, happy, long | 4.43 (3.88) | 4.02 | 0.00 | | 100.00 | 7.15 | |
| Comparatives | greater, best, after | 1.41 (1.64) | 1.24 | 0.00 | | 100.00 | 17.76 | |
| Interrogatives | how, when, what | 2 (2.26) | 1.67 | 0.00 | | 100.00 | 12.48 | |
| Numbers | second, thousand | 0.44 (1.05) | 0.26 | 0.00 | | 100.00 | 34.19 | |
| Quantifiers | few, many, much | 1.68 (1.88) | 1.49 | 0.00 | | 100.00 | 14.45 | |
| **Affective Processes** | happy, cried | 8.94 (6.33) | 8.17 | 0.00 | | 100.00 | 4.14 | |
| Positive emotion | love, nice, sweet | 6.81 (6.03) | 5.77 | 0.00 | | 100.00 | 5.19 | |
| Negative emotion | hurt, ugly, nasty | 2.1 (2.43) | 1.75 | 0.00 | | 100.00 | 14.65 | |
| Anxiety | worried, fearful | 0.15 (0.58) | 0.00 | 0.00 | | 50.00 | 55.85 | |
| Anger | hate, kill, annoyed | 0.81 (1.37) | 0.43 | 0.00 | | 50.86 | 31.70 | |
| Sadness | crying, grief, sad | 0.52 (1.21) | 0.30 | 0.00 | | 100.00 | 31.95 | |
| **Social Words** | mate, talk, they | 13.22 (6.83) | 12.74 | 0.00 | | 100.00 | 2.99 | |
| Family words | daughter, dad, aunt | 1.27 (2.32) | 0.73 | 0.00 | | 100.00 | 20.18 | |
| Friends | buddy, neighbor | 0.43 (1.1) | 0.18 | 0.00 | | 100.00 | 37.92 | |
| Female references | girl, her, mom | 1.38 (2.27) | 0.90 | 0.00 | | 100.00 | 20.62 | |
| Male references | boy, his, dad | 1.46 (2.1) | 1.02 | 0.00 | | 100.00 | 18.84 | |
| **Cognitive Processes** | cause, know, ought | 8.98 (4.58) | 9.28 | 0.00 | | 100.00 | 5.39 | |
| Insight | think, know | 1.45 (1.42) | 1.33 | 0.00 | | 57.15 | 17.15 | |
| Causation | because, effect | 1.46 (1.79) | 1.30 | 0.00 | | 100.00 | 15.75 | |
| Discrepancies | should, would | 1.71 (1.59) | 1.59 | 0.00 | | 100.00 | 15.02 | |
| Tentativeness | maybe, perhaps | 2.12 (1.75) | 2.00 | 0.00 | | 54.55 | 13.49 | |
| Certainty | always, never | 1.22 (1.53) | 1.01 | 0.00 | | 100.00 | 19.59 | |
| Differentiation | hasn’t, but, else | 2.29 (1.9) | 2.20 | 0.00 | | 100.00 | 13.95 | |
| **Perceptual Processes** | look, hear, feeling | 2.91 (3.12) | 2.57 | 0.00 | | 100.00 | 9.44 | |
| Seeing | view, saw, seen | 1.31 (2.22) | 0.95 | 0.00 | | 100.00 | 17.37 | |
| Hearing | listen, hearing | 0.76 (1.59) | 0.55 | 0.00 | | 100.00 | 24.56 | |
| Feeling | feels, touch | 0.67 (1.41) | 0.44 | 0.00 | | 100.00 | 28.25 | |
| **Biological Processes** | eat, blood, pain | 3.69 (4.05) | 3.04 | 0.00 | | 100.00 | 8.74 | |
| Body | cheeks, hands, spit | 1.1 (1.71) | 0.76 | 0.00 | | 50.00 | 22.52 | |
| Health or illness | clinic, flu, pill | 0.81 (1.98) | 0.40 | 0.00 | | 100.00 | 27.90 | |
| Sexuality | horny, love, incest | 0.45 (1.89) | 0.05 | 0.00 | | 100.00 | 48.29 | |
| Ingesting | dish, eat, pizza | 0.93 (2.02) | 0.50 | 0.00 | | 100.00 | 27.02 | |
| **Core Drives and Needs** | friend, win, danger | 8.6 (5.15) | 8.14 | 0.00 | | 100.00 | 3.77 | |
| Affiliation | ally, friend, social | 2.72 (3.35) | 2.14 | 0.00 | | 100.00 | 10.38 | |
| Achievement | win, success, better | 1.13 (1.54) | 0.93 | 0.00 | | 100.00 | 18.97 | |
| Power | superior, bully | 2.21 (2.94) | 1.80 | 0.00 | | 100.00 | 11.14 | |
| Reward focus | take, prize, benefit | 2.49 (2.26) | 2.23 | 0.00 | | 100.00 | 10.65 | |
| Risk or prevention focus | danger, doubt | 0.47 (1.36) | 0.27 | 0.00 | | 100.00 | 32.50 | |
| **Time Orientation** | | | | | | | | |
| Focus on the past | ago, did, talked | 3.68 (2.67) | 3.61 | 0.00 | | 100.00 | 9.89 | |
| Focus on the present | today, is, now | 12.23 (5.36) | 12.50 | 0.00 | | 100.00 | 3.75 | |
| Focus on the future | may, will, soon | 1.81 (1.93) | 1.62 | 0.00 | | 100.00 | 14.02 | |
| **Relativity** | area, bend, exit | 14.8 (6.19) | 14.84 | 0.00 | | 100.00 | 2.91 | |
| Motion | arrive, car, go | 2.87 (2.67) | 2.65 | 0.00 | | 100.00 | 9.56 | |
| Space | down, in, thin | 6.75 (4.17) | 6.49 | 0.00 | | 100.00 | 5.12 | |
| Time | end, until, season | 5.57 (3.65) | 5.33 | 0.00 | | 100.00 | 6.14 | |
| **Personal Concerns** | | | | | | | | |
| Work | job, major, xerox | 1.62 (2.77) | 1.14 | 0.00 | | 100.00 | 16.38 | |
| Leisure | cook, chat, movie | 1.25 (2.43) | 0.81 | 0.00 | | 100.00 | 19.43 | |
| Home | kitchen, landlord | 0.92 (1.94) | 0.58 | 0.00 | | 100.00 | 22.37 | |
| Money | audit, cash, owe | 1 (1.99) | 0.59 | 0.00 | | 100.00 | 23.28 | |
| Religion | altar, church | 0.5 (1.67) | 0.11 | 0.00 | | 100.00 | 42.12 | |
| Death | bury, coffin, kill | 0.13 (0.67) | 0.00 | 0.00 | | 66.67 | 63.78 | |
| **Informal Speech** |  | 6.49 (6.37) | 5.42 | 0.00 | | 100.00 | 6.26 | |
| Expletives | damn, shit | 0.94 (1.66) | 0.37 | 0.00 | | 50.86 | 35.43 | |
| Netspeak | btw, lol, thx | 2.51 (3.65) | 1.68 | 0.00 | | 100.00 | 16.75 | |
| Assent | agree, ok, yes | 2.28 (4.45) | 1.45 | 0.00 | | 100.00 | 13.51 | |
| Nonfluencies | er, hm, umm | 0.46 (1.2) | 0.22 | 0.00 | | 100.00 | 37.25 | |
| Fillers | Imean, youknow | 0.08 (0.3) | 0.00 | 0.00 | | 25.00 | 71.00 | |
